# First Evidence of Siglec-10 Localization and Expression in Camel Male Reproductive Tissues and Spermatozoa: Potential Relevance to Fertility

**DOI:** 10.3390/vetsci12111063

**Published:** 2025-11-06

**Authors:** Fatemah Alzilaiy, Marwa Babiker, Khalid ALkhodair

**Affiliations:** Department of Anatomy, College of Veterinary Medicine, King Faisal University, P.O. Box 400, Al-Hassa 31982, Saudi Arabia; mbabakr@kfu.edu.sa (M.B.); kalkhodair@kfu.edu.sa (K.A.)

**Keywords:** Siglec-10, *Camelus dromedarius*, testis, epididymis, spermatozoa

## Abstract

Siglec-10 is a cell-surface lectin that belongs to the sialic acid-binding immunoglobulin-like lectins (Siglecs) receptor involved in immune tolerance, but its role in reproduction remains unknown. Because immune regulation is essential for sperm survival and fertilization, Siglec-10 may represent an unexplored factor in camel fertility. Here, we investigated its expression and localization in the male reproductive tract and spermatozoa of dromedary camels during the rutting season. Testis, epididymis, and vas deferens were analyzed by immunohistochemistry and quantitative PCR, while spermatozoa from fresh ejaculates, frozen–thawed semen, and epididymal samples were examined using chromogenic and fluorescent immunostaining. Siglec-10 was strongly expressed in the testis and epididymis but absent in the vas deferens. In spermatozoa, the signal was localized mainly to the head and midpiece, with consistent patterns in fresh and frozen–thawed semen but absent in epididymal sperm. These findings provide the first descriptive evidence of Siglec-10 in camel reproductive tissues and ejaculated spermatozoa, suggesting a possible involvement in mechanisms that support sperm integrity under stress conditions; however, further studies are needed to clarify its functional role in semen preservation, and highlighting its relevance as a fertility biomarker in seasonal breeders.

## Simple Summary

Camels are seasonal breeders, and their fertility is often influenced by environmental and physiological factors. Understanding the molecules that protect sperm and support reproduction is important for improving breeding programs. In this study, we examined Siglec-10, a protein usually known for its role in the immune system, in the male reproductive tract and sperm of dromedary camels. We found that Siglec-10 was present in the testis and epididymis but absent in the vas deferens. On sperm, it was detected only in ejaculated sperm (fresh and frozen–thawed) but not in epididymal sperm. Its specific localization to the acrosome, neck, midpiece, and annulus, together with its persistence after freezing, suggests that Siglec-10 may help sperm survive and remain functional. These findings offer preliminary insights into camel reproduction and may inform future research on semen preservation and assisted reproductive strategies.

## 1. Introduction

Male fertility relies on a delicate balance between protective immunity and tolerance within the male reproductive tract (MRT). Because most germ-cell antigens appear only after puberty, they bypass central immune tolerance, leaving the testis vulnerable to autoimmune attack [1]. To safeguard spermatogenesis, the testis establishes an immune-privileged state through structural barriers, such as the blood–testis barrier (BTB), and local immunoregulatory mediators that suppress excessive immune responses [2]. Sertoli cells are central to this regulation, combining barrier formation with tolerogenic signaling, yet they can also mount antimicrobial defenses when required [3]. Similarly, the epididymis must tolerate sperm while remaining capable of pathogen defense, with regional variations in immune sensitivity; the cauda, for instance, is more susceptible to inflammatory injury than the caput [4,5]. Disruption of these mechanisms by infection, chronic inflammation, or aging impairs sperm production and function, ultimately compromising male fertility [6,7].

The sialic-acid binding immunoglobulin-like lectins (Siglecs) are a family of cell surface receptors that recognize sialylated glycans and act as regulators of immune cell communication [8,9]. Many members contain immunoreceptor tyrosine-based inhibitory motifs (ITIMs) that help maintain immune homeostasis [10,11]. Siglec-10, in particular, is broadly expressed on human leukocytes and functions as an inhibitory receptor by binding to ligands such as CD24 and phosphatidylserine, thereby promoting tolerance and limiting inflammatory activation [12,13,14]. Despite these well-defined immunological roles, the distribution of Siglec-10 outside the immune system remains poorly understood, and its presence in the male reproductive tract or spermatozoa has not yet been investigated.

Siglec family members have been extensively studied in humans and experimental animals, mainly in the context of immune cell biology [8]. Siglec-10 has been linked to immune tolerance, with altered signaling associated with chronic inflammation, autoimmunity, and tumor immune evasion [12,13,15]. Several Siglec homologues have also been identified in mice, cattle, and pigs, reflecting their evolutionary conservation [16,17]. However, data on Siglecs in reproductive organs remain scarce. While some isoforms have been detected on human spermatozoa and within the female reproductive tract [18,19], the presence and distribution of Siglec-10 in the male reproductive system or spermatozoa of any species have not yet been described.

The dromedary camel (*Camelus dromedarius*) is an economically and culturally important livestock species in arid regions, and its reproductive performance is strongly shaped by seasonal breeding patterns [20]. Several molecular markers, such as aquaporins and hormone receptors, have been described in the male reproductive tract of this species [21,22], but information on immunoregulatory molecules remains scarce. In particular, no data exist on the expression or localization of Siglec-10 in camel reproductive tissues or spermatozoa. This knowledge gap limits our understanding of how immune-modulatory pathways may influence sperm survival, fertility, and semen preservation in this seasonal breeder.

Interestingly, Siglec-10 is known to interact with ligands such as CD24 and phosphatidylserine, both of which are associated with “don’t-eat-me” signals involved in immune tolerance and cell survival. Given that spermatozoa, particularly after ejaculation, may be exposed to maternal immune surveillance, it is plausible that Siglec-10 contributes to sperm immune evasion or protection from immune-mediated clearance. Furthermore, one could hypothesize that the presence of Siglec-10 may help preserve sperm integrity under stress conditions such as inflammation or cryopreservation, where immune activation can impair sperm function.

This study was designed to provide the first descriptive evidence of Siglec-10 expression in the male reproductive tract and spermatozoa of the dromedary camel. Using chromogenic and fluorescence immunohistochemistry together with qRT-PCR, we mapped the localization and transcript profile of this inhibitory receptor in testis, epididymis, vas deferens, and spermatozoa collected during the rutting season. Our findings demonstrate that Siglec-10 is expressed in camel reproductive tissues and ejaculated sperm but absent from epididymal sperm. This novel observation extends the biology of Siglec-10 beyond its established immune context and opens new perspectives for reproductive immunology. Importantly, the persistence of Siglec-10 in frozen–thawed spermatozoa highlights its potential as a fertility biomarker and as a target for semen preservation strategies, providing a foundation for improving assisted reproductive technologies in seasonal breeders.

## 2. Materials and Methods

### 2.1. Ethical Statement

All animal procedures were conducted in accordance with institutional guidelines and national regulations of the Kingdom of Saudi Arabia governing animal slaughter and research ethics. The animal study protocol was reviewed and approved by the Research Ethics Committee at King Faisal University (KFU-REC; Approval No. KFU-REC-2024-JUN-ETHICS1899). Tissue sampling was performed at a licensed abattoir in Al-Ahsa from animals slaughtered for commercial purposes; no animal was euthanized specifically for research. Semen collections were carried out under ketamine–xylazine anesthesia at the Camel Research Center (King Faisal University) and supervised by a licensed veterinarian, with all efforts made to minimize discomfort.

#### 2.1.1. Experimental Animals and Tissue Collection

Testes, epididymides, and vas deferens were collected from thirty (30) clinically healthy adult male dromedary camels (*Camelus dromedarius*) of the indigenous breed, aged 5–15 years, during the rutting season (November–March) at a licensed abattoir in Al-Ahsa, Saudi Arabia. Postmortem examination confirmed the absence of pathological alterations in the reproductive organs. Immediately after excision, surrounding connective tissue and smooth muscle layers were carefully removed.

For immunohistochemical (IHC) analysis, tissue fragments were obtained from the cranial (T.C), caudal (T.Ca), and rete testis (R.T); from the caput (E.H), corpus (E.B), and cauda (E.T) segments of the epididymis; and from the vas deferens (V.D). Samples were fixed in 10% neutral-buffered formalin (Sigma-Aldrich, St. Louis, MO, USA) for 36 h. For molecular assays, additional tissue specimens were preserved overnight at 4 °C in RNAlater^®^ solution (Thermo Fisher Scientific, Waltham, MA, USA), trimmed into ~50 mg fragments, placed into 2.0 mL screw-cap tubes, and stored at −80 °C until RNA extraction for qRT-PCR analysis.

#### 2.1.2. Immunohistochemistry for Siglec-10 Detection

Immunohistochemical assays were performed on testicular, epididymal, and deferential tissues collected from thirty adult camels (*n* = 30 biological replicates). For each animal, three non-consecutive sections were analyzed per tissue (*n* = 3 technical replicates).

Paraffin-embedded tissue blocks were sectioned at 5 μm and mounted onto Superfrost™ Plus charged glass slides (Thermo Scientific, Waltham, MA, USA). Sections were deparaffinized in xylene and rehydrated through a descending ethanol series, followed by rinsing in phosphate-buffered saline (PBS, pH 7.4; Sigma-Aldrich, St. Louis, MO, USA). Antigen retrieval was performed in 10 mM sodium citrate buffer (pH 6.0) by microwave heating (20 min). After cooling to room temperature (25 °C), slides were washed three times in Tris-buffered saline containing 0.1% Tween-20 (TBST). Endogenous peroxidase activity was quenched with 3% hydrogen peroxide (H_2_O_2_) for 10 min, and non-specific binding was blocked with 10% normal goat serum for 10 min.

Primary antibody incubation was carried out overnight (14 h) at 4 °C in a humidified chamber using rabbit polyclonal anti-Siglec-10 primary antibody (Molecule-On, Auckland New Zealand; Cat. No. AB-M-134; dilution 1:200). On the following day, slides were rinsed in TBST and incubated for 30 min at room temperature with goat anti-rabbit IgG secondary antibody (Abcam, Cambridge, UK; Cat. No. ab64261). Signal amplification was achieved with a streptavidin–horseradish peroxidase (HRP) complex, and visualization was performed with 3,3′-diaminobenzidine (DAB) for 5 min. Sections were counterstained with hematoxylin, dehydrated in graded ethanol, cleared in xylene, and mounted with dibutylphthalate polystyrene xylene (DPX; Sigma-Aldrich). Negative controls were included by replacing the primary antibody with normal rabbit IgG Table 1.

Digital micrographs were captured using a Leica DM6000B microscope equipped with a Leica DFC420 camera (Leica Microsystems, Wetzlar, Germany) at 20× and 40× magnifications, and images were processed with LAS V4.2 software. Immunolabeling intensity was assessed using two complementary approaches: (1) semi-quantitative analysis with Fiji ImageJ software (version 1.53t, NIH, Bethesda, MD, USA) to calculate integrated optical density (IOD) and stained area fraction [23]; and (2) independent visual scoring by three observers, classifying staining intensity as strong (+++), moderate (++), weak (+), or absent (−).

##### RNA Isolation and cDNA Preparation

Total RNA was extracted from homogenized testicular, epididymal, and deferential tissues using the PureZOL™ RNA isolation reagent (Bio-Rad, Hercules, CA, USA; Cat. No. 732-6890). To eliminate potential genomic DNA contamination, samples were treated with DNase I (Ambion DNase I Kit, Thermo Fisher Scientific, Waltham, MA, USA; Cat. No. AM2222) according to the manufacturer’s instructions.

RNA concentration and purity were assessed by spectrophotometric analysis (A260/280 ratio) using a BioTek Synergy HTX microplate reader (BioTek Instruments, Winooski, VT, USA). From each sample, 1 μg total RNA was reverse transcribed into complementary DNA (cDNA) using the iScript™ Advanced cDNA Synthesis Kit (Bio-Rad, Hercules, CA, USA; Cat. No. 170-8843) in a 20 μL reaction volume, following the supplier’s protocol.

Reverse transcription was performed under the following thermal profile: 25 °C for 10 min, 42 °C for 15 min, 85 °C for 5 min, followed by a final hold at 4 °C. The resulting cDNA was stored at −20 °C until further use in qRT-PCR analysis.

##### Quantitative Real-Time PCR (qRT-PCR)

Quantitative real-time PCR was performed on cDNA synthesized with the iScript™ Advanced cDNA Synthesis Kit (Bio-Rad, Hercules, CA, USA; Cat. No. 170-8843). Each amplification reaction contained 1 μL of cDNA template, gene-specific primers, and iTaq™ Universal SYBR^®^ Green Supermix (Bio-Rad, Hercules, CA, USA; Cat. No. 172-5121) in a 20 μL final volume. Primer sets specific for Siglec-10 and the reference gene GAPDH were designed using the NCBI Primer-BLAST tool (http://www.ncbi.nlm.nih.gov/tools/primer-blast/, accessed on 18 September 2025), and their sequences are listed in Table 2.

Thermal cycling conditions were as follows: initial denaturation at 95 °C for 10 min, followed by 40 cycles of denaturation at 95 °C for 5 s, annealing at 65 °C for 10 s, and extension at 72 °C for 20 s. All reactions were performed in triplicate to ensure reproducibility.

Relative expression levels of Siglec-10 were calculated using the 2^−ΔΔCT^ method, with GAPDH as the endogenous control for normalization. Analyses were based on RNA samples from thirty camels (*n* = 30 biological replicates), with three technical replicates included per gene and per sample (*n* = 3).

### 2.2. Sperm Collection and Immunostaining

#### 2.2.1. Fresh Ejaculated Sperm

Fresh ejaculated semen was collected from six mature, fertile dromedary camels during the rutting season using an electroejaculator (ELECTROJAC6, Anicam Enterprises Inc., Springfield, MO, USA) under ketamine–xylazine anesthesia at the Camel Research Center, King Faisal University (Al-Ahsa, Saudi Arabia). All procedures were conducted in compliance with approved animal welfare guidelines. Undiluted semen was transported to the laboratory within 1 h in a 37 °C insulated container.

#### 2.2.2. Frozen–Thawed Sperm

Frozen semen straws were obtained from the Camel Research Center (King Faisal University) and stored in liquid nitrogen. Straws were thawed gradually at room temperature, and spermatozoa were washed to remove extender components prior to immunostaining.

#### 2.2.3. Epididymal Sperm

Epididymal spermatozoa were collected postmortem from the cauda epididymis of 30 mature camels at a local abattoir during the rutting season. A small incision was made in the epididymal tail, and released spermatozoa were aspirated into sterile tubes containing phosphate-buffered saline (PBS), as previously described.

#### 2.2.4. Sample Preparation

All sperm samples were washed twice in PBS by centrifugation at 2000 rpm for 5 min, and pellets were resuspended in PBS. A 15 µL aliquot was smeared onto Superfrost^®^ slides (Thermo Fisher Scientific, Waltham, Massachusetts, USA), air-dried, and stored at −20 °C until use.

#### 2.2.5. Fluorescent Immunostaining

Slides were circled with a hydrophobic barrier pen and rinsed in PBS. Non-specific binding was blocked with 1:20 normal rabbit serum (Sigma-Aldrich, St. Louis, MO, USA) for 20 min at room temperature. After rinsing, slides were incubated for 1 h at room temperature with rabbit polyclonal anti-Siglec-10 primary antibody (Molecule-On, New Zealand; Cat. No. AB-M-134; dilution 1:200). A FITC-conjugated goat anti-rabbit IgG secondary antibody (Abcam, Cambridge, UK; Cat. No. ab64261) was applied for 2 h at room temperature in the dark. After three PBS washes, coverslips were mounted with antifade medium containing DAPI (Vector Laboratories, Burlingame, CA, USA).

#### 2.2.6. Chromogenic Immunostaining

Parallel smears were processed for chromogenic detection. After PBS rinses, endogenous peroxidase activity was quenched with 3% hydrogen peroxide (H_2_O_2_; Sigma-Aldrich) for 10 min. Non-specific binding was blocked with 1:20 normal goat serum (Sigma-Aldrich) for 20 min, followed by incubation with rabbit polyclonal anti-Siglec-10 antibody (Molecule-On, Auckland, New Zealand; Cat. No. AB-M-134; dilution 1:200) for 1 h at room temperature. Slides were washed and incubated with an HRP-conjugated goat anti-rabbit IgG secondary antibody (Abcam, Cambridge, UK; Cat. No. ab64261) for 30 min, followed by avidin–biotin complex (Vector Laboratories, Burlingame, CA, USA) for 20 min. Visualization was performed with 3,3′-diaminobenzidine (DAB; Sigma-Aldrich) for 5 min, counterstaining with hematoxylin, dehydration in graded ethanol, and mounting with DPX (Sigma-Aldrich).

#### 2.2.7. Negative Controls and Visualization

Negative controls were prepared by omitting the primary antibody and replacing it with goat anti-rabbit IgG secondary antibody (Abcam, Cambridge, UK; Cat. No. ab64261). For fluorescence assays, stained slides were examined under a Leica DM6000B fluorescence microscope equipped with a Leica DFC420 camera (Leica Microsystems, Wetzlar, Germany) using FITC and DAPI filter sets. For chromogenic assays, slides were evaluated under a Leica DM6000B bright-field microscope (Leica Microsystems, Wetzlar, Germany). Representative digital images were captured at 40× and 100× magnifications and processed using LAS V4.2 software (Leica Microsystems, Wetzlar, Germany).

### 2.3. Statistical Analysis

All datasets were analyzed using one-way analysis of variance (ANOVA), followed by Tukey’s post hoc test for multiple comparisons to assess significant differences among groups. Statistical analyses were performed using GraphPad Prism software (version 9.5.1, San Diego, CA, USA). Results are expressed as mean ± standard error of the mean (SEM). A probability value of *p* < 0.05 was considered statistically significant. Each experimental group included thirty biological replicates (*n* = 30), and all measurements were conducted in technical triplicates to ensure reproducibility.

## 3. Results

### 3.1. Immunohistochemical Detection of Siglec-10 in Tissues

In the male reproductive tract immunohistochemical staining demonstrated that Siglec-10 was differentially distributed across the male reproductive tract of dromedary camels during the rutting season. Clear positive signals were detected in all examined regions of the testis and epididymis, with staining intensity varying between segments. In contrast, no immunolabeling was observed in the vas deferens. These observations were verified independently by three evaluators and were further confirmed by semi-quantitative measurements in ImageJ (64-bit), which assessed both integrated optical density and percentage of stained area (Table 3).

#### 3.1.1. Testes

**Figure 1:**
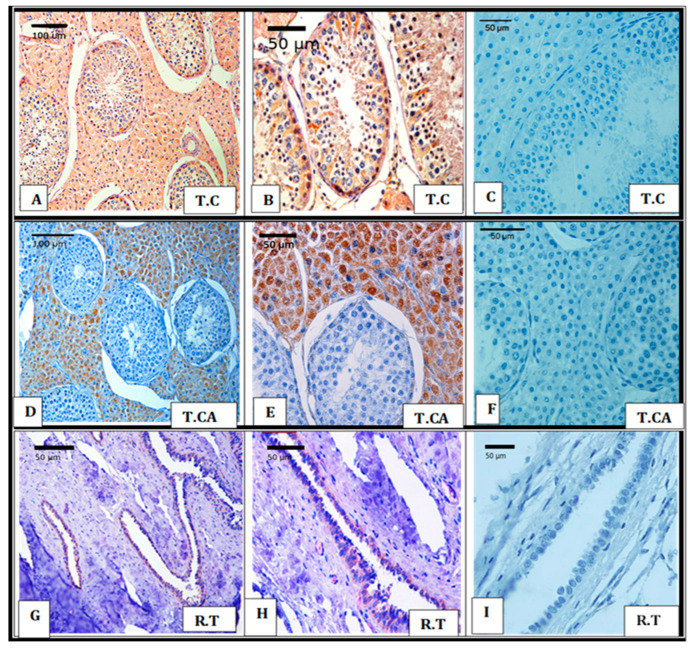
Chromogen-based immunohistochemical detection of Siglec-10 in the cranial region (T.C), caudal region (T.Ca), and rete testis (R.T) of dromedary camels during the breeding season. (**A**,**D**,**G**) Low-magnification images (20× objective) show positive cytoplasmic labeling in Leydig cells of T.C and T.Ca, as well as weak cytoplasmic reactivity in the epithelial lining of the R.T. (**B**,**E**,**H**) Higher-magnification images (40× objective) demonstrate cytoplasmic immunolabeling for Siglec-10 in spermatogonia, secondary spermatocytes, late spermatids, and Sertoli cells within the seminiferous tubules, in addition to strong labeling in Leydig cells. (**C**,**F**,**I**) Negative control sections confirm the specificity of the staining. Immunoreactivity was visualized using DAB chromogen, followed by hematoxylin counterstaining.

In both the cranial and caudal regions of the testis, Leydig cells within the intertubular spaces displayed strong cytoplasmic staining for Siglec-10, while no nuclear labeling was detected, indicating that the protein was confined to the cytoplasm (Figure 1A,D,E). Within the seminiferous tubules, spermatogenic cells—including spermatogonia, secondary spermatocytes, and late spermatids—as well as Sertoli cells, also showed clear cytoplasmic positivity (Figure 1B). In contrast, mature spermatozoa located in the tubular lumen lacked detectable staining, confirming the absence of Siglec-10 in fully differentiated germ cells (Figure 1A). The epithelial lining of the rete testis exhibited only weak cytoplasmic staining, again without nuclear labeling (Figure 1G,H). A summary of staining patterns and intensities across testicular regions is provided in Table 3.

#### 3.1.2. Epididymis

**Figure 2:**
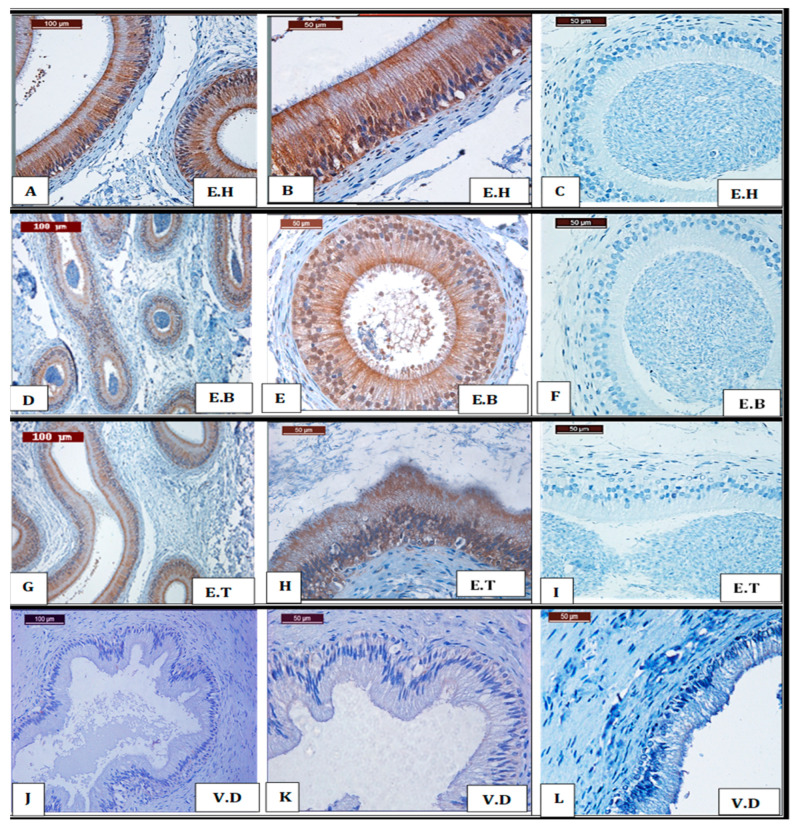
Chromogen-based immunohistochemical evaluation of Siglec-10 in the head (E.H), body (E.B), and tail (E.T) of the epididymis, as well as the vas deferens, during the breeding season. (**A**,**D**,**G**) Low-magnification images (20× objective) show distinct immunolabeling in the epithelial lining cells of the epididymal regions. (**B**,**E**,**H**) Higher-magnification images (40× objective) demonstrate pronounced cytoplasmic immunostaining in the epithelial lining of the epididymal head (E.H) and tail (E.T), whereas the body region (E.B) displayed only moderate cytoplasmic signals. (**J**,**K**) No detectable labeling for Siglec-10 was observed in any structural component of the vas deferens under either low or high magnification. (**C**,**F**,**I**,**L**) Negative control sections confirmed staining specificity. Immunoreactivity was visualized using DAB chromogen, followed by hematoxylin counterstaining.

In the caput region of the epididymis, the pseudostratified epithelium showed strong Siglec-10 staining, particularly in principal and basal cells, with cytoplasmic signals evident in both apical and basal compartments (Figure 2A,B). No immunolabeling was detected in the stereocilia. In the corpus, epithelial cells exhibited moderate cytoplasmic staining, mainly localized to the apical domain (Figure 2E), while stereocilia showed only faint reactivity and luminal spermatozoa remained negative (Figure 2D). In the cauda, immunostaining intensity increased again, with dense cytoplasmic labeling in the epithelial lining, but stereocilia appeared short and did not display specific reactivity (Figure 2G,H). Across all regions, spermatozoa within the lumen lacked detectable signals. The summarized distribution and intensity of immunostaining in each epididymal segment are presented in Table 3.

#### 3.1.3. Vas Deferens

In the vas deferens, immunohistochemical examination revealed a complete lack of Siglec-10 labeling. Both low- and high-power views confirmed the absence of specific staining, with no cytoplasmic or nuclear reactivity detected in any of the epithelial or stromal cell types (Figure 2J,K).

#### 3.1.4. Semi-Quantitative Analysis of Siglec-10 Immunoreactivity in the Testis and Epididymis of the Dromedary Camel

**Figure 3:**
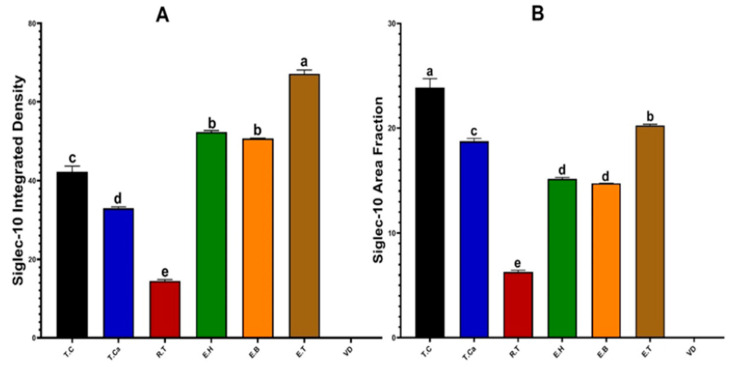
Semi-quantitative analysis of Siglec-10 immunoreactivity in the male reproductive system of Camelus dromedarius during the breeding season. (**A**) Integrated optical density of immunostaining in testicular regions, epididymal segments, and the vas deferens. (**B**) Area fraction (%) of immunostaining within the same tissues. Data are expressed as mean ± SEM (*n* = 30). Different superscript letters (a, b, c, d, e) indicate statistically significant differences among tissue types (*p* < 0.05). Abbreviations: T.C, cranial testis; T.Ca, caudal testis; R.T, rete testis; E.H, epididymal head; E.B, epididymal body; E.T, epididymal tail; V.D, vas deferens.

In the testis, the caudal region exhibited a significantly lower integrated optical density (33.00 ± 0.36) compared with the cranial region (42.25 ± 1.44; *p* < 0.05), but remained significantly higher than that of the rete testis (14.55 ± 0.34; *p* < 0.05) (Figure 3A). Among all testicular regions, the cranial part displayed the highest area fraction (23.90 ± 0.84; *p* < 0.05), whereas the rete testis showed the lowest (6.31 ± 0.15; *p* < 0.05). The caudal region presented an intermediate value (18.75 ± 0.28), which differed significantly from both the cranial and rete testis (*p* < 0.05) (Figure 3B).

In the epididymis, the tail region demonstrated the highest integrated optical density (67.22 ± 0.97; *p* < 0.05) across all examined regions of the male reproductive tract. The head and body segments displayed comparable values (52.33 ± 0.39 and 50.71 ± 0.09, respectively), both significantly greater than those observed in testicular regions (*p* < 0.05). Regarding the area fraction, the epididymal tail also exhibited the highest value (20.24 ± 0.12; *p* < 0.05), significantly greater than the head (15.17 ± 0.13) and body (14.74 ± 0.16), which did not differ significantly from each other.

Quantitative analysis revealed significant regional differences in Siglec-10 expression along the male reproductive tract (Figure 3). Elevated expression levels were observed in the cranial and caudal regions of the testis, where Leydig and Sertoli cells are prominent. Strong immunoreactivity was also detected in the tail of the epididymis. In contrast, no detectable expression was observed in the vas deferens. These spatial expression patterns suggest differential involvement of Siglec-10 in local tissue environments.

#### 3.1.5. qRT-PCR Analysis

**Figure 4:**
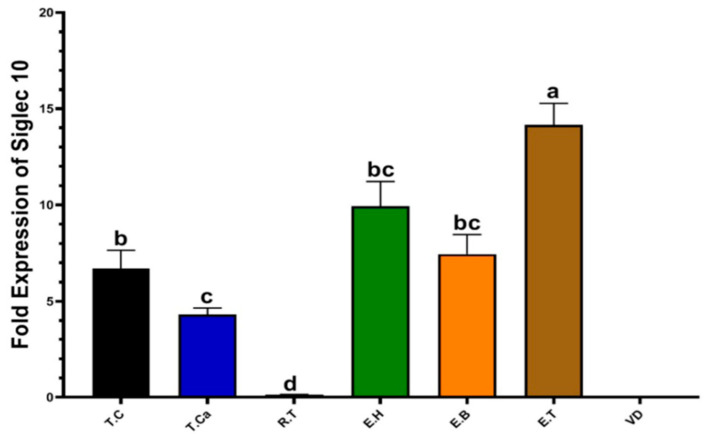
Relative mRNA expression of Siglec-10 in the male reproductive tract of Camelus dromedarius during the rutting season, quantified by qRT-PCR and normalized to GAPDH. Results are expressed as mean ± SEM (*n* = 30). Different superscript letters (a, b, c, d) indicate statistically significant differences among tissues (*p* < 0.05).

To further evaluate the molecular regulation of Siglec-10, quantitative real-time polymerase chain reaction (qRT-PCR) was performed. Figure 4 illustrates Siglec-10 mRNA transcription levels across different regions of the male reproductive tract (MRT) in dromedary camels. No expression of Siglec-10 mRNA was detected in the vas deferens. By contrast, distinct differences were observed among testicular and epididymal tissues.

Within the testis, the cranial region exhibited significantly higher mRNA expression (6.70 ± 0.90; *p* < 0.05) compared with both the caudal region (4.31 ± 0.30) and the rete testis (0.14 ± 0.01). In the epididymis, the tail showed markedly elevated expression (14.16 ± 1.14; *p* < 0.05), significantly greater than the head (9.94 ± 1.28) and body (7.46 ± 1.01). Collectively, these findings demonstrate that the cranial testis and epididymal tail are the principal sites of Siglec-10 mRNA transcription during the rutting season.

Our qRT-PCR analysis revealed significant regional variation in Siglec-10 transcript levels across the male reproductive tract. Elevated expression was observed in the cranial testis and epididymal tail, both corresponding to sites of active spermatogenesis and sperm maturation. This indicates that Siglec-10 transcription is most pronounced in regions associated with germ cell development. By contrast, no detectable expression was found in the vas deferens, suggesting a lack of transcriptional activity during the later stages of sperm transport. These findings highlight a region-specific pattern of Siglec-10 gene expression within the male reproductive system of dromedary camels.

### 3.2. Siglec-10 Detection in Camel Spermatozoa

Below (Table 4), we present the detailed findings regarding the presence and regional distribution of Siglec-10 across differ-ent sperm domains.

#### 3.2.1. Immunostaining of Fresh Camel Sperm with Siglec-10 Antibody

**Figure 5:**
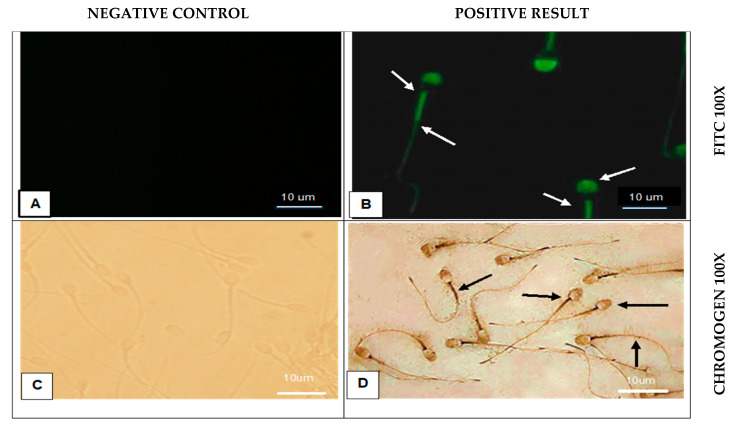
Immunostaining of camel fresh sperm with Siglec-10 antibody. (**A**) Negative control (−Ve), no primary antibody (No 1 Ab), FITC-conjugated antibodies (FITC), 100×. (**B**) Positive staining (+Ve), Siglec-10, FITC, 100×. (**C**) (−Ve), (No 1 Ab), with chromogenic stain (Chromogen), 100×. (**D**) (+Ve), Siglec-10, Chromogen, 100×. Arrows indicate positive staining.

To assess the localization of Siglec-10 in fresh ejaculated camel spermatozoa, both chromogenic and fluorescent immunostaining techniques were employed. The results were compared with negative controls to confirm the specificity of the antibody signal (Figure 5).

#### 3.2.2. Immunostaining of Frozen Camel Sperm with Siglec-10 Antibody

**Figure 6:**
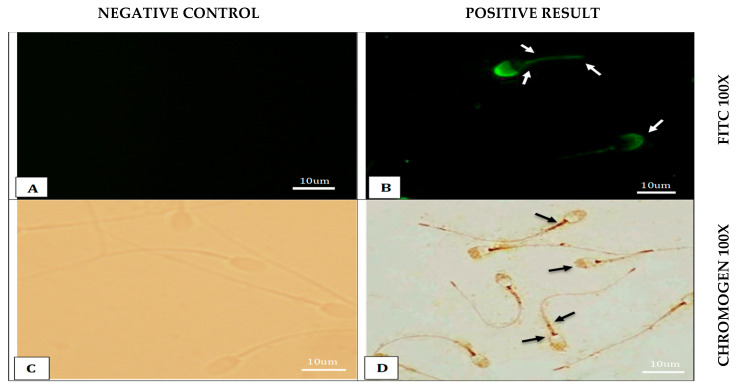
Immunostaining of camel frozen sperm with Siglec-10 antibody. (**A**) Negative control (−Ve), no primary antibody (No 1 Ab), FITC-conjugated antibodies (FITC), 100×. (**B**) Positive staining (+Ve), Siglec-10, FITC, 100×. (**C**) (−Ve), (No 1 Ab), with chromogenic stain (Chromogen), 100×. (**D**) (+Ve), Siglec-10, Chromogen, 100×. Arrows indicate positive staining.

To evaluate whether the cryopreservation process affects the localization of Siglec-10, frozen–thawed camel spermatozoa were examined using both FITC-conjugated fluorescent and chromogenic immunostaining techniques. Control samples without primary antibody were included to ensure staining specificity. The results below demonstrate the patterns of Siglec-10 expression in frozen sperm samples (Figure 6).

#### 3.2.3. Immunostaining of Epididymal Camel Sperm with Siglec-10 Antibody

**Figure 7:**
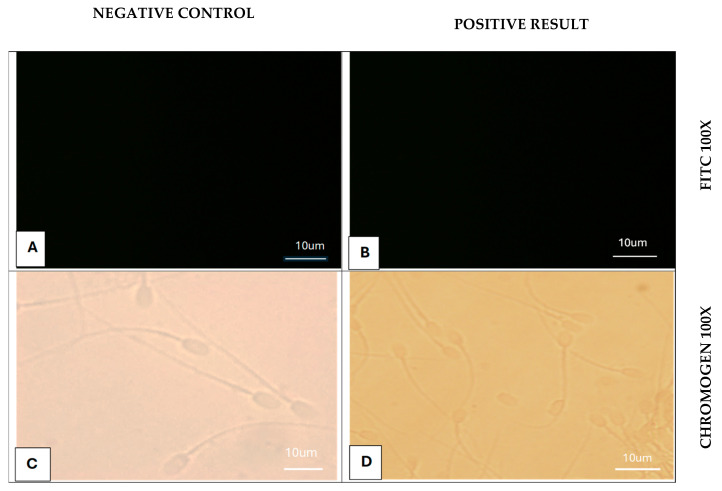
Immunostaining of camel Epididymal sperm with Siglec-10 antibody. (**A**) Negative control (−Ve), no primary antibody (No 1 Ab), FITC-conjugated antibodies (FITC), 100×. (**B**) Positive staining (+Ve), Siglec-10, FITC, 100×. (**C**) (−Ve), (No 1 Ab), with chromogenic stain (Chromogen), 100×. (**D**) (+Ve), Siglec-10, Chromogen, 100×.

Immunostaining of camel spermatozoa revealed that Siglec-10 is expressed in fresh and frozen–thawed sperm, while epididymal sperm showed no detectable staining (Table 4, Figure 5, Figure 6 and Figure 7). Positive immunoreactivity was restricted to the acrosomal cap, neck, midpiece, and annulus, whereas the equatorial band, post-acrosomal cap, and tail were negative. No staining was observed in the negative controls, confirming the specificity of the signal. Both chromogenic and fluorescent detection methods gave comparable results, with fluorescence providing higher resolution. Importantly, no variation was detected between fresh and frozen sperm, suggesting that cryopreservation did not alter Siglec-10 expression.

## 4. Discussion

This study provides the first descriptive evidence that Siglec-10 is expressed in the male reproductive tract of the dromedary camel. Immunohistochemistry and qRT-PCR confirmed its presence in the testis and epididymis, whereas no detectable expression was found in the vas deferens. Immunostaining of spermatozoa further revealed localization in ejaculated sperm—both fresh and frozen–thawed—but not in epididymal sperm. The signal was compartmentalized to the acrosomal cap, neck, midpiece, and annulus, with consistent persistence following cryopreservation. Together, these findings establish a tissue- and domain-specific distribution of Siglec-10 in camels and add a novel dimension to the molecular landscape of the male reproductive system [12,18,19].

In the testis, Siglec-10 immunoreactivity was detected in Sertoli cells, spermatogonia, and Leydig cells, suggesting that this receptor may contribute to the local immune regulatory milieu. The testis is a well-established immune-privileged organ, where the blood–testis barrier and Sertoli-cell-derived factors help protect germ cells from autoimmune responses while preserving the ability to mount antimicrobial defenses when challenged [1,3]. The presence of an inhibitory receptor such as Siglec-10 in both somatic and germ cell compartments adds another layer of regulation that could help maintain tolerance against developing germ cells, many of which express novel antigens not represented during central tolerance [24]. Similar patterns have been described for other immunoregulatory molecules in rodent and human testes, supporting the idea that immune checkpoint pathways are active in this tissue [6,25]. Given that altered immune regulation in the testis has been associated with orchitis, impaired spermatogenesis, and infertility [5], the detection of Siglec-10 here points to a potential role in maintaining immune balance essential for reproductive capacity.

In the epididymis, Siglec-10 immunoreactivity was evident in the epithelial principal cells along the caput, corpus, and cauda, with regional variation in intensity. This observation is consistent with the concept that the epididymis is not only a site of sperm maturation and storage but also an immunologically active organ [26]. The epithelium must balance tolerance toward spermatozoa, which are antigenically “foreign” to the host, with effective protection against pathogens. Recent studies have highlighted that this immune landscape is compartmentalized, with the cauda being particularly vulnerable to inflammation and tissue damage [27,28]. The detection of Siglec-10 in principal cells suggests that inhibitory signaling may be involved in maintaining this balance, potentially preventing excessive immune responses that could damage sperm while preserving the ability to recognize harmful stimuli. Comparable mechanisms have been described for other inhibitory molecules, such as PD-L1, in the epididymis of rodents and humans, where they help sustain immune tolerance in the luminal environment [29,30].

In the vas deferens, Siglec-10 immunostaining was completely absent in the epithelial lining, in contrast to its clear presence in the testis and epididymis. Epithelial cells of the vas deferens are known to contribute to mucosal immunity through antimicrobial peptides and inflammatory mediators [31,32], but the lack of Siglec-10 expression suggests that other regulatory pathways predominate in this terminal segment. This negative result highlights the tissue-specific nature of Siglec-10 distribution in the camel reproductive system and implies that its functional role is likely confined to germ cell development and sperm maturation rather than sperm transport.

A striking observation of this study was the detection of Siglec-10 on ejaculated spermatozoa, both fresh and frozen–thawed, whereas epididymal sperm were consistently negative. The receptor localized specifically to the acrosomal cap, neck, midpiece, and annulus, but was absent from the equatorial band, post-acrosomal region, and tail. This compartmentalized distribution suggests that Siglec-10 may influence sperm functions dependent on these domains, including acrosome stability, mitochondrial activity, and head–tail coupling [33,34]. The absence of Siglec-10 in epididymal sperm, together with its presence in ejaculated cells, indicates that it may be acquired during ejaculation, possibly through interactions with accessory gland secretions or seminal plasma proteins [35]. Notably, the persistence of Siglec-10 following cryopreservation is significant, since freezing and thawing often alter membrane proteins and compromise sperm viability [36]. Its stability may therefore reflect a role in maintaining membrane integrity or in modulating immune recognition after insemination. Similar findings for other Siglec family members in human sperm have suggested potential involvement in fertilization and immune evasion within the female tract [21].

This study provides the first demonstration of Siglec-10 expression in the camel male reproductive tract and ejaculated spermatozoa. This specific localization pattern may imply a role in protecting sperm structure during physiological transitions such as transport and activation. Its detection after freezing and thawing could be indicative of stability, but functional implications remain to be investigated in future studies. These results introduce a novel molecular candidate of practical relevance for camel fertility, pointing to Siglec-10 as a promising biomarker for semen quality and preservation in seasonal breeders. Although the present work is primarily descriptive, a notable strength lies in the relatively large sample size (*n* = 30), which is uncommon in camel reproductive research and enhances the statistical robustness and reliability of our findings. Future studies should now address its mechanistic roles and evaluate its potential application in assisted reproduction.

## 5. Conclusions

This study provides the first evidence of Siglec-10 expression in the male reproductive tract and ejaculated spermatozoa of the dromedary camel. The receptor was localized in Sertoli, Leydig, and germ cells of the testis, in principal cells of the epididymis, and on specific sperm domains (acrosomal cap, neck, midpiece, and annulus), but absent in the vas deferens and epididymal sperm. The persistence of Siglec-10 following cryopreservation raises the hypothesis of a potential relevance to sperm resilience and storage, warranting further investigation to determine its exact role in assisted reproductive settings. These findings extend the biological significance of Siglec-10 beyond immune cells and suggest its promise as a fertility-associated biomarker with relevance to assisted reproduction in seasonal breeders.

## Figures and Tables

**Table 1:** Primary and Secondary Antibodies Used for Immunohistochemistry.

Antibody/Kit	Host Species	Company	Catalog No.	Application	Dilution
Rabbit polyclonal anti-Human Siglec-10	Rabbit	Molecule-On, Auckland, New Zealand	AB-M-134	IHC	1:200
Rabbit specific HRP/DAB (ABC) Detection IHC Kit (includes biotinylated goat anti-rabbit IgG (H + L), streptavidin–HRP, protein block, H_2_O_2_ block, and DAB substrate/chromogen)	Goat (secondary)	Abcam, Cambridge, UK	ab64261	IHC	As per kit proto

**Table 2:** Primer sequences used for the quantitative real-time PCR analysis of Siglec-10 and GAPDH gene expression in dromedary camels.

Gene Name	Primer Sequence (5′→3′)	Accession Number
Siglec-10	F-ACGCCTCCTACATGGTCAAC R-ACTTCTTGGGAACTCCGCTG	XM 064489724.1
GAPDH	F-CCTGGAGAAACCTGCCAAATA R-CTATTGAAGTCGCAGGAGACAA	EU331417.1

**Table 3:** Semi-quantitative evaluation of Siglec-10 immunostaining intensity, expressed as integrated optical density (IOD), across the male reproductive tract of dromedary camels (Camelus dromedarius).

Tissue Sample							
Staining Intensity	Testis			Epididymis			Vas Deferens
	TC	TCa	RT	EH	EB	ET	VD
Strong	*+++*	*+++*		*+++*		*+++*	
Moderate					*++*		
Weak			*+*				
Absent							−

Abbreviations: TC, cranial region of the testis; TCa, caudal region of the testis; RT, rete testis; EH, epididymal head; EB, epididymal body; ET, epididymal tail; VD, vas deferens. Staining intensity scores: (−) absent; (+) weak; (++) moderate; (+++) strong.

**Table 4:** Siglec-10 distribution on fresh, frozen and epidydimal sperm from camels.

Sample Type							
	AC	EB	PAC	N	MP	A	T
Fresh sperm	+	-	-	+	+	+	-
Frozen sperm	+	-	-	+	+	+	-
Epididymal sperm	-	-	-	-	-	-	-

(+) Positive; (-) Negative; (AC) Acrosomal Cap; (EB) Equatorial Band; (PAC) Post-Acrosomal Cap; (N) Neck; (MP) Midpiece; (A) Annulus; (T) Tail.

## Data Availability

The original contributions presented in this study are included in the article. Further inquiries can be directed to the corresponding author.
